# Cholangioscopy-assisted endoscopic transpapillary gallbladder drainage serves as an alternative treatment for an inoperable patient with gallbladder rupture

**DOI:** 10.1055/a-2888-0464

**Published:** 2026-06-25

**Authors:** Hsiang Wen Cho, Ming-Chang Tsai, Edy Kornelius, Chao-Yen Huang, Hon Phin Wong, Chi-Chih Wang

**Affiliations:** 1 Division of Gastroenterology, Department of Internal Medicine 63276 Chung Shan Medical University HospitalTaichung CityTaiwan; 2 School of Medicine 34899 Chung Shan Medical UniversityTaichung CityTaiwan; 3 Division of Endocrinology and Metabolism, Department of Internal Medicine 63276 Chung Shan Medical University HospitalTaichung CityTaiwan; 4 Emergency Medicine 63276 Chung Shan Medical University HospitalTaichung CityTaiwan; 5 Department of Surgery 63276 Chung Shan Medical University HospitalTaichung CityTaiwan

A 94-year-old man presented with septic shock and severe abdominal pain. His underlying diseases were three-vessel coronary artery disease, treated with stent imp-lantation, with severe heart failure, paroxysmal atrial fibrillation receiving anticoagulation agents, chronic obstructive pulmonary disease and previous cholangitis status after cholangiopancreatography for common bile duct stones.

**Fig. 1:**
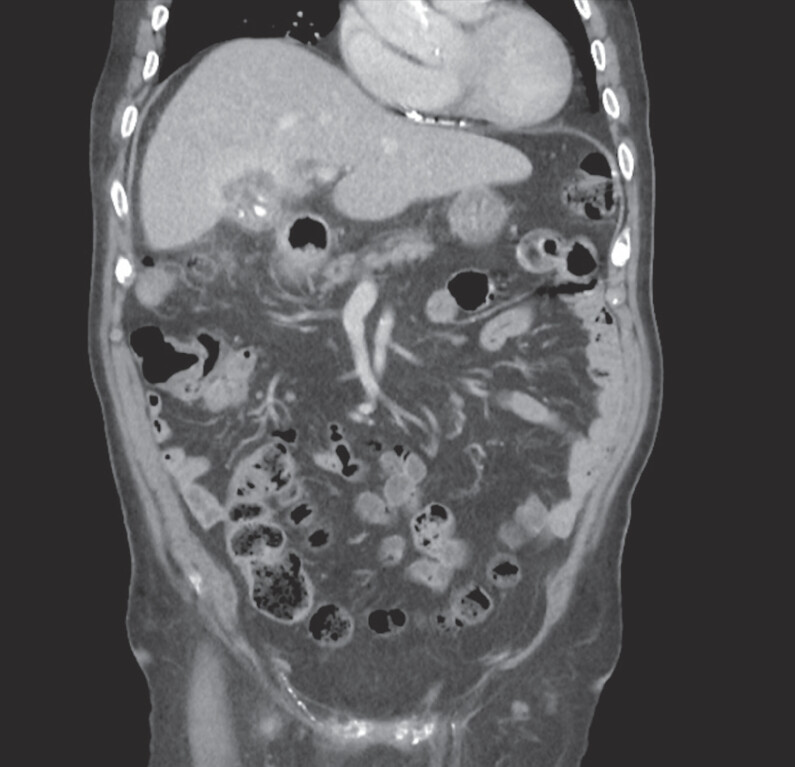
An abdominal computed tomography revealed acute cholecystitis complicated with gallbladder rupture.

This time, abdominal computed tomography (CT) revealed acute cholecystitis complicated with gallbladder rupture ( Fig. 1 ). Initial management followed guideline-recommended conservative treatment, 1​ with percutaneous transhepatic gallbladder drainage (PTGBD).

**Fig. 2:**
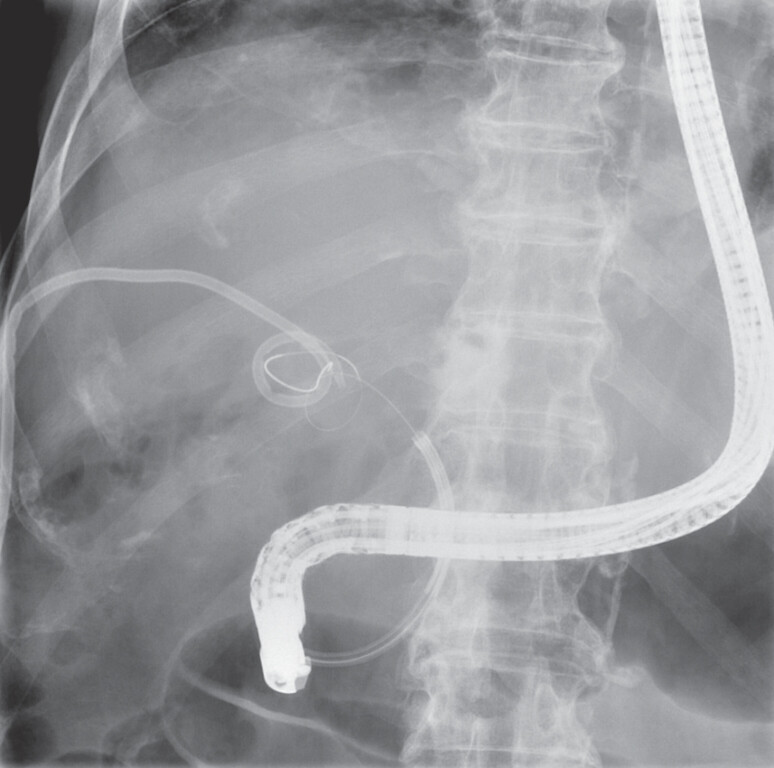
ETGBD was performed using a duodenoscope with a SpyGlass Direct Visualization System, which enabled direct visualization and selected wire cannulation into ruptured gallbladder.

**Fig. 3:**
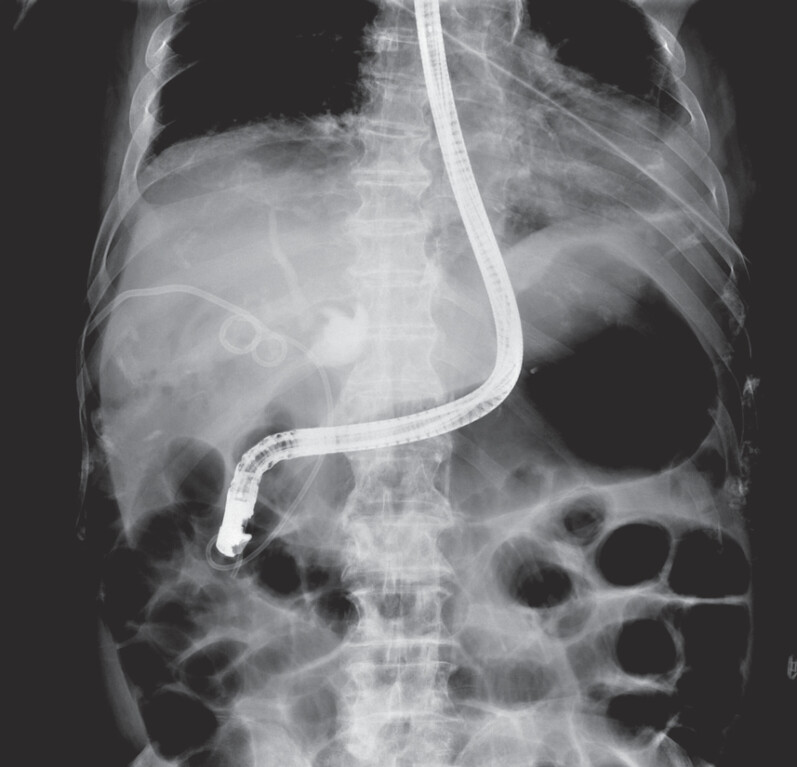
Internal drainage was successfully established using a double pigtailed drainage tube.

**Fig. 4:**
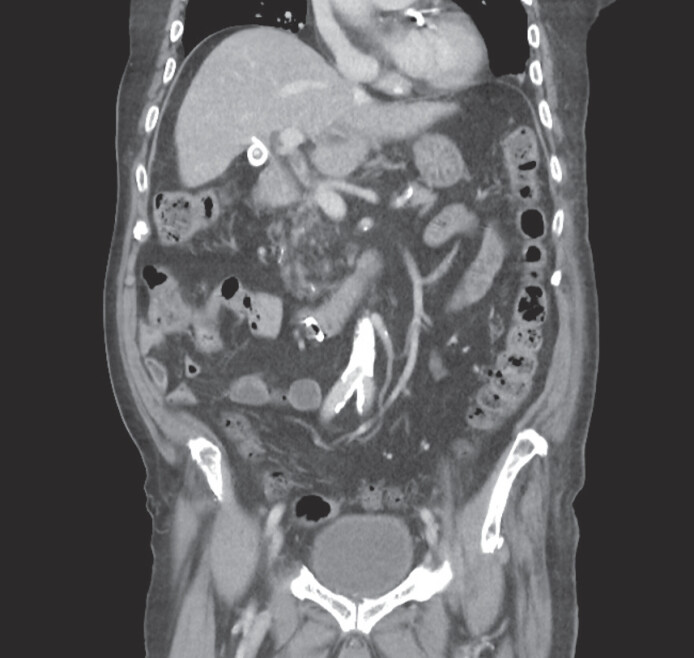
A contrast enhanced CT demonstrated the shrinkage and disappearance of the original gallbladder with ETGBD at the same location 2 years later.

To avoid prolonged dependence on external catheter drainage and provide durable internal gallbladder decompression, endoscopic transpapillary gallbladder drainage (ETGBD) was subsequently planned. ETGBD was performed using a duodenoscope with a single-operator cholangioscopy system ( Fig. 2 and Video 1 ), which enabled the direct visualization and clear identification of the cystic duct orifice. Internal drainage was successfully established using a double pigtailed drainage tube ( Fig. 3 ). Following the confirmation of effective internal drainage, the previously placed PTGBD catheter was removed. The patient remained clinically stable and a CT demonstrated the shrinkage and disappearance of the original gallbladder with ETGBD at the same location 2 years later ( Fig. 4 ).

**Video 1** Cholangioscopy-assisted ETGBD for definitive internal drainage in an inoperable gallbladder rupture patient.


Current guidelines recommend gallbladder drainage for patients with severe acute cholecystitis, such as gallbladder rupture when surgery is not feasible. 2​ However, they provide limited guidance regarding subsequent long-term management in patients who are permanently inoperable. In recent years, endoscopic gallbladder drainage has emerged as an alternative to PTGBD in high-risk patients. 3​ ​4​ This case highlights the feasibility and safety of a step-up approach from PTGBD to ETGBD in an elderly patient with substantial comorbidity and gallbladder rupture. Cholangioscopy-assisted ETGBD allowed the precise identification of the cystic duct and reliable guidewire access into the gallbladder, 5​ enabling effective internal drainage and eliminating the need for prolonged external catheterization. Our case represents a practical non-surgical option for durable gallbladder decompression with long-term internal drainage in patients with gallbladder rupture.

Endoscopy_UCTN_Code_TTT_1AR_2AZ
